# The Structure-Activity Relationship between Marine Algae Polysaccharides and Anti-Complement Activity

**DOI:** 10.3390/md14010003

**Published:** 2015-12-24

**Authors:** Weihua Jin, Wenjing Zhang, Hongze Liang, Quanbin Zhang

**Affiliations:** 1 Key Laboratory of Experimental Marine Biology, Institute of Oceanology, Chinese Academy of Sciences, Qingdao 266071, China; jinweihua@qdio.ac.cn (W.J.); wenjingwing@126.com (W.Z.); 2 College of Earth Science, University of Chinese Academy of Sciences, Beijing 100049, China; 3 Laboratory for Marine Biology and Biotechnology, Qingdao National Laboratory for Marine Science and Technology, Qingdao 266071, China; 4 The School of Materials Sciences and Chemical Engineering, Ningbo University, Ningbo 315211, China; lianghongze@nbu.edu.cn

**Keywords:** marine algae polysaccharides, anti-complement activity, structure-activity relationship

## Abstract

In this study, 33 different polysaccharides were prepared to investigate the structure-activity relationships between the polysaccharides, mainly from marine algae, and anti-complement activity in the classical pathway. Factors considered included extraction methods, fractionations, molecular weight, molar ratio of galactose to fucose, sulfate, uronic acid (UA) content, linkage, branching, and the type of monosaccharide. It was shown that the larger the molecular weights, the better the activities. The molar ratio of galactose (Gal) to fucose (Fuc) was a positive factor at a concentration lower than 10 µg/mL, while it had no effect at a concentration more than 10 µg/mL. In addition, sulfate was necessary; however, the sulfate content, the sulfate pattern, linkage and branching had no effect at a concentration of more than 10 µg/mL. Moreover, the type of monosaccharide had no effect. Laminaran and UA fractions had no activity; however, they could reduce the activity by decreasing the effective concentration of the active composition when they were mixed with the active compositions. The effect of the extraction methods could not be determined. Finally, it was observed that sulfated galactofucan showed good anti-complement activity after separation.

## 1. Introduction

The complement system is an important part of the innate immune system that is designed to eliminate “harmful” substances from the body. This elimination is accomplished in five different ways according to a previous study [1]. Inappropriate activation of the complementary target self-tissues causes pathology in a large number of inflammatory, ischaemic, and other diseases [2].

There is a large body of research on naturally-occurring complement inhibitors isolated from animal, plant, and microbial products, such as polysaccharides [3,4,5,6], phenolic acid [7], proteins [8], flavonoids, and steroides [9,10]. Many synthetic molecules, including dextran sulfate [11,12], nafamastat mesilate (FUT-175) [2] and compstatin [1] have been shown to inhibit activation of the complement system. In addition, many studies have also reported [13,14,15] that a sulfated polysaccharide from brown algae, has been found to inhibit complement activation. Structure and activity studies showed that sulfated polysaccharides are potential candidates for screening as inhibitors of the complement system. Marine algae contain numerous different polysaccharides, which are non-toxic. In addition, they also contain polyanions and are majorly substituted by sulfate, which differs from the polysaccharides from land-plants.

Currently, marine algae mainly contain brown algae, red algae, and green algae. Polysaccharides from brown algae [16,17,18,19,20,21] contain three major types of polysaccharides, namely, fucoidan, laminaran, and alginate. Fucoidan includes sulfated heteropolysaccharides and sulfated galactofucan or fucan. The former contains sulfated glucuronomannan, glucuronan, galactan, *etc.* Sulfated glucuronomannan has a backbone of alternating 2-linked mannopyranose residue and 4-linked glucuronic acid, sulfated at C6 of the mannnopyranose residue. The linkage of glucuronan is 3-linked. Galactan has a backbone of 6-linked galactopyranose residue branched at C4 with galactose, while the latter one is made up of 3-linked fucopyranose residues or alternating 3-linked and 4-linked fucopyranose residues. The major differences between sulfated galactofucan or fucan are the positions of the sulfate and branching units, including the fucose residues, galactose residues, glucuronic acid residues, and so on. Laminaran is a glucan, consisting mainly of 3-linked glucopyranose residue, branched with 6-linked glucopyranose. Alginate contains polymannuronic acid, polyguluronic acid and a mixture of polymannuronic acid and polyguluronic acid. Polysaccharides from red algae [18,21,22,23,24] contain three types of polysaccharides, namely, carrageenan polysaccharides, agar polysaccharides, and an agar-carrageenan intermediate polysaccharide. They have a backbone of alternating 3-linked β-d-galactopyranose and 4-linked α-galactopyranose residues; however, carrageenan polysaccharides have 4-linked α-d-galactopyranose residues while agar polysaccharides have 4-linked α-l-galactopyranose residues. In addition, all the α-galactopyranose residues might exist in the form of 3,6-substituted by ester sulfate, methyl groups, pyruvic acid acetal, *etc.* Water-soluble polysaccharides from green algae [18,23,25] can be divided into two types of polysaccharides. Type 1 is classified as xylogalactoarabian, which mainly consists of xylose, galactose, and arabinose. It contains a backbone of 4-linked or 5-linked arabinopyranose residues sulfated at C3, 3-linked or 6-linked galactopyranose residues sulfated at C4 or C6, and 4-linked xylopyranose residues. Type 2 is named as glucuronoxylorhamnan, which mainly contains glucuronic acid, xylose, and rhamnose, according to the type of major monosaccharides. It consists of 4-linked rhamnopyranose sulfated at C3 or C2, a 4-linked glucuronic acid residue and a 4-linked xylopyranose residue.

To clarify the structure-activity relationship between marine algae polysaccharides and their anti-complement activity, several types of polysaccharides from brown algae, red algae, and green algae were prepared. In addition, many factors including the extraction methods, fractionations, molecular weight, the molar ratio of galactose to fucose, sulfate, UA content, linkage, branching, and the type of monosaccharides were taken into account.

## 2. Results and Discussion

### 2.1. The Effect of the Extraction Methods on the Anti-Complement Activity

Table 1 displays the chemical composition of two fucoidans, which were derived from *Saccharina japonica* by hot water (SJW) and diluted hydrochloric acid (SJS). It was shown that the contents of fucose (Fuc) and sulfate contained in SJS were higher than in SJW, while the content of UA contained in SJS was lower. In addition, the molecular weight of SJS was also lower because of degradation during the process of extraction by dilute hydrochloric acid. Moreover, it also indicated that SJW contained a greater number of other monosaccharides, suggesting that the structure of SJW was more complex than that of SJS. In terms of the chemical compositions of HFW (HFW was obtained from brown algae *Hizikia fusiforme* by hot water) and the HFS (HFS was obtained from brown algae *Hizikia fusiforme* by dilute hydrochloric acid), as shown in Table 1, Fuc, UA and the molecular weight of the HFW were higher than in HFS, while the sulfate content of HFW was lower. The molar ratios of monosaccharides indicated that both HFS and HFW contained substantial amounts of laminaran, which was also reported in a previous study [26]. To summarize, different extraction methods might influence the chemical compositions of the polysaccharides.

**Table 1:** Chemical composition (%, dry weight) of the polysaccharides studied.

Sample	Yields (%)	Fuc (%)	UA (%)	SO4 (%)	Monosaccharides (Molar Ratio)							Mw (kDa)
					Man	Rha	GlcA	Glc	Gal	Xyl	Fuc	
SJW	1.21	22.38	10.32	30.60	0.10	0.03	0.15	0	0.63	0.07	1	152.4
SJW-1	10.67	15.84	33.36	15.05	0.55	0.10	0.96	0	1.22	0.37	1	77.8
SJW-2	19.99	17.01	17.35	28.33	0.12	0.03	0.18	0	1.08	0.10	1	88.4
SJW-3	37.89	28.96	0.45	35.10	0.02	0	0.02	0	0.27	0	1	162.7
SJS	1.08	34.97	3.44	36.88	0.06	0.03	0.06	0.01	0.11	0.02	1	106.3
SJS-1	8.98	21.56	13.71	19.75	0.06	0.02	0.06	0.02	0.17	0.02	1	63.8
SJS-2	15.89	31.07	8.21	34.84	0.08	0.03	0.07	0.01	0.28	0.03	1	84.5
SJS-3	40.78	36.94	0	38.43	0.01	0	0.01	0	0.05	0	1	125.4
HFW	2.43	28.04	5.43	28.46	0.11	0	0.08	0.62	0.27	0.09	1	118.3/3.9
HFW-1	18.51	-	-	-	-	-	-	1	-	-	-	3.9
HFW-2	18.16	22.78	17.64	17.33	0.48	0.30	0.27	0.29	0.23	0.42	1	116.7/5.1
HFW-3	28.01	32.85	0	31.62	0.05	0.01	0.04	0.01	0.29	0.04	1	114.2
HFS	1.53	21.89	1.98	30.59	0.07	0.01	0.09	1.22	0.25	0.02	1	97.3/3.6
HFS-1	16.24	-	-	-	-	-	-	1	-	-	-	3.4
HFS-2	10.66	25.79	20.84	25.66	0.27	0.02	0.20	0.03	0.22	0.04	1	42.0/4.3
HFS-3	9.72	40.84	0.98	39.85	0	0	0	0	0.27	0.01	1	99.2
SJS-OS	98.19	33.31	3.31	46.36	0.06	0.03	0.05	0.05	0.20	0.05	1	119.5
SJS-DS	65.41	41.20	6.11	18.12	0.05	0.03	0.05	0.03	0.18	0.05	1	117.5/33.4
SJS-DS-OS	110.1	24.24	3.79	35.30	0.05	0.02	0.04	0.03	0.20	0.05	1	123.6
ANW		25.08	4.16	27.36	0.05	0.03	0.09	0.29	0.09	0.06	1	122.1
SC	5.78	21.80	6.54	28.15	0.15	0.32	0.13	0.22	0.28	0.08	1	111.9
SJW-2-R	38.99	18.56	9.86	29.63	0.10	0.03	0.06	0.10	1.05	0.07	1	79.6
SJW-2-HJ	10.89	14.35	29.36	14.63	0.46	0.10	0.50	0	2.10	0.20	1	50.4
SJW-2-HW	83.10	25.23	8.36	35.69	0.09	0.01	0.10	0	0.56	0.06	1	95.6
HFW-1-S	109.1	-	-	41.38	-	-	-	1	-	-	-	13.7
ES	23.19	-	-	26.33	-	-	-	-	1	-	-	147.9
GF	13.30	-	-	20.42	-	-	-	-	1	-	-	137.2
GL	30.28	-	-	2.74	-	-	-	-	1	-	-	146.0
PY	10.71	-	-	2.72	-	-	-	-	1	-	-	140.7
EP	19.53	-	26.77	18.09	-	1	0.37	0.13	0.06	0.31	-	189.3
UP	18.75		28.39	20.88	-	1	0.45	0.15	0.04	0.33	-	163.4
CR	8.13	-	-	32.84	-	0.03	-	-	0.14	1	-	174.9
CF	7.89	-	-	23.54	-	0.05	-	0.40	1	0.50	-	173.4

Polysaccharides derived from *Saccharina japonica* by hot water (SJW) and dilute hydrochloric acid (SJS). SJW was fractionated by anion exchange chromatography into three components, namely SJW-1, SJW-2 and SJW-3. SJS was also fractionated by anion exchange chromatography into three components, namely SJS-1, SJS-2 and SJS-3. Polysaccharides derived from *Hizikia fusiforme* by hot water (HFW) and dilute hydrochloric acid (HFS). HFW was fractionated by anion exchange chromatography into three components, namely HFW-1, HFW-2 and HFW-3. HFS was also fractionated by anion exchange chromatography into three components, namely HFS-1, HFS-2 and HFS-3. Overly sulfated SJS (SJS-OS) and laminaran (HFW-1-S), desulfated SJS (SJS-DS) and desulfated SJS with sulfation (SJS-DS-OS). Polysaccharides derived from *Ascophyllum nodosum* (ANW), *Acaudina molpadioides* (SC), *Eucheuma spinosum* (ES), *Grateloupia filicina* (GF), *Gracilaria lamaneiformis* (GL), *Porphyra yezoensis* (PY), *Enteromorpha prolifera* (EP), *Ulva pertusa* (UP), *Cladophera rupestris* (CR) and *Codium fragile* (CF). SJW-2-R was obtained through UA reduction, and SJW-2-HJ and SJW-2-HW were fractionated using an activated carbon column.

**Figure 1:**
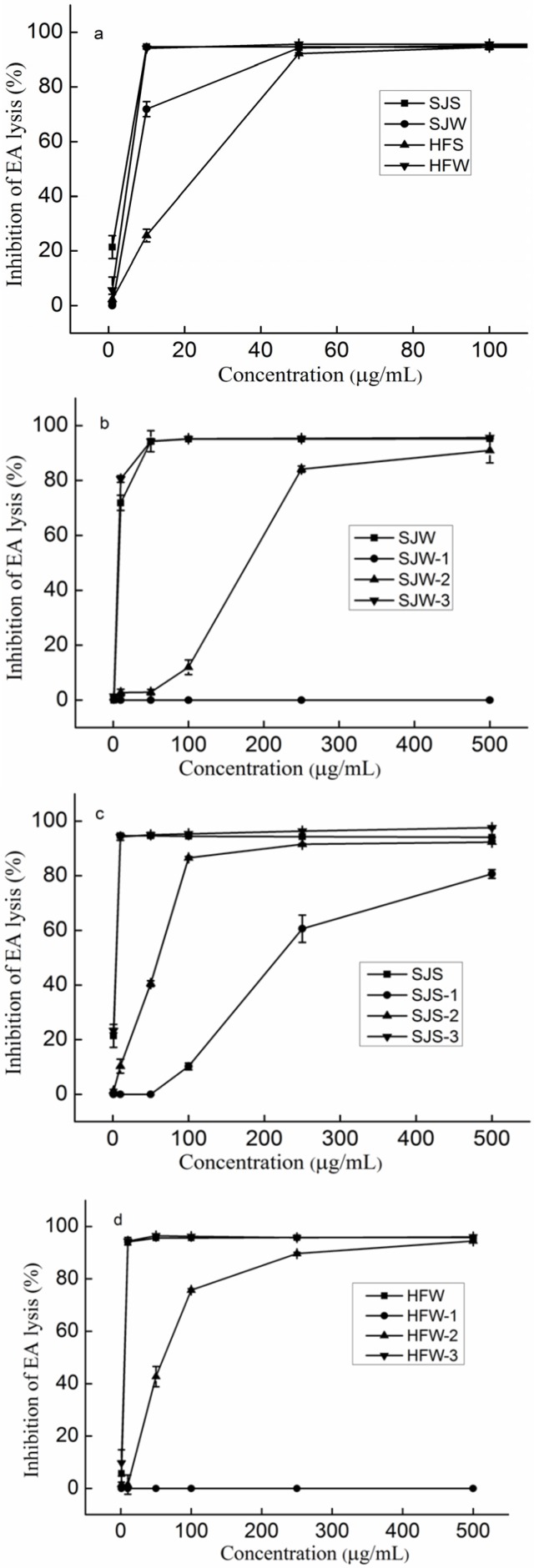

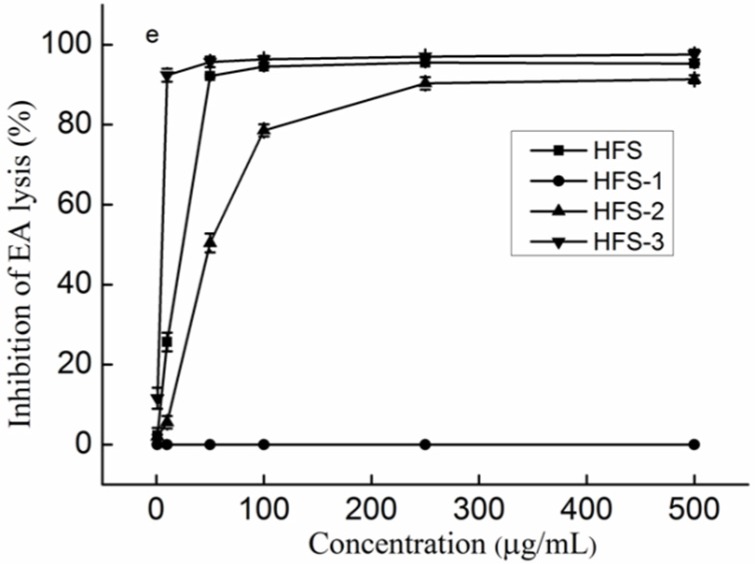
Inhibition of the classical pathway-mediated haemolysis of EA in 1:10-diluted NHS in the presence of increasing amounts of the polysaccharides (**a**) SJS, SJW, HFS, and HFW; (**b**) SJW and its fractions; (**c**) SJS and its fractions; (**d**) HFW and its fractions; (**e**) HFS and its fractions. The results are expressed as percent inhibition of haemolysis. Data are the means from three determinations ± S.E.M. Polysaccharides derived from *Saccharina japonica* by hot water (SJW) and dilute hydrochloric acid (SJS). SJW was fractionated by anion exchange chromatography into three components, namely SJW-1, SJW-2 and SJW-3. SJS was also fractionated by anion exchange chromatography into three components, namely SJS-1, SJS-2 and SJS-3. Polysaccharides derived from *Hizikia fusiforme* by hot water (HFW) and dilute hydrochloric acid (HFS). HFW was fractionated by anion exchange chromatography into three components, namely HFW-1, HFW-2 and HFW-3. HFS was also fractionated by anion exchange chromatography into three components, namely HFS-1, HFS-2 and HFS-3.

Figure 1a displays the anti-complement activities of SJS, SJW, HFS and HFW in the classical pathway. Activities of SJS and HFW reached a plateau at a concentration of 10 µg/mL, while SJW and HFS reached a plateau at a concentration of 50 µg/mL. Specifically, the IC_50_ of SJS, SJW, HFS, and HFW were 4.51, 7.26, 24.65, and 5.51 µg/mL, respectively. Thus, it was difficult to determine the effects of the extraction methods because the IC_50_ of SJS was lower than that of SJW, while the IC_50_ of HFW was lower than that of HFS.

### 2.2. The Effect of the Fractionations on the Anti-Complement Activity

SJW was fractionated by anion exchange chromatography into three components, namely, SJW-1, SJW-2, and SJW-3. Chemical analysis indicated that SJW-1 had the highest UA content and SJW-3 had the highest fucose content and sulfate content. In addition, SJW-3 had the highest molecular weight. Previous studies [27,28,29] reported that SJW-3 was mainly sulfated galactofucan, and SJW-1 and SJW-2 were sulfated heteropolysaccharides. Figure 1b shows the anti-complement activities of SJW and its fractions. Compared to SJW and its fractions, it was found that the activity of SJW-3 was similar to the SHW activity, while SJW-2 had lower activity and SJW-1 had no activity. Specifically, the IC_50_ of SJW-3 (3.11 µg/mL) was lower than that of SJW (7.26 µg/mL). The chemical composition of SJS’s fractions (SJS was fractionated by anion exchange chromatography into three components, namely, SJS-1, SJS-2, and SJS-3) were similar to the results of SJW’s fractions, suggesting that SJS-3 was a sulfated galactofucan, while SJS-1 and SJS-2 were sulfated heteropolysaccharides. The activities of SJS and its fractions in Figure 1c confirmed the above results. HFW was also fractionated by anion exchange chromatography into three components, namely, HFW-1, HFW-2, and HFW-3. HFW-1 was composed of glucose, HFW-2 had plenty of the other monosaccharides and HFW-3 contained galactose and fucose. According to the chemical compositions in Table 1, it was proposed that HFW-1 was mainly laminaran, HFW-2 was mainly sulfated heteropolysaccharides, and HFW-3 was mainly sulfated galactofucan, which was in accordance with a previous study [26]. Figure 1d also shows that HFW-3 had the best activity. HFS was fractionated by anion exchange chromatography into three components, namely, HFS-1, HFS-2, and HFS-3. Table 1 showed that HFS-1 contained only glucose, HFS-2 had many other monosaccharides, while HFS-3 had galactose and fucose, suggesting that they were similar to the fractions of HFW. The activities of HFS and its fractions in Figure 1e also confirmed the above results. In conclusion, sulfated galactofucan contributed to the anti-complement activities.

### 2.3. The Effect of Molecular Weight on the Anti-Complement Activity

**Figure 2:**
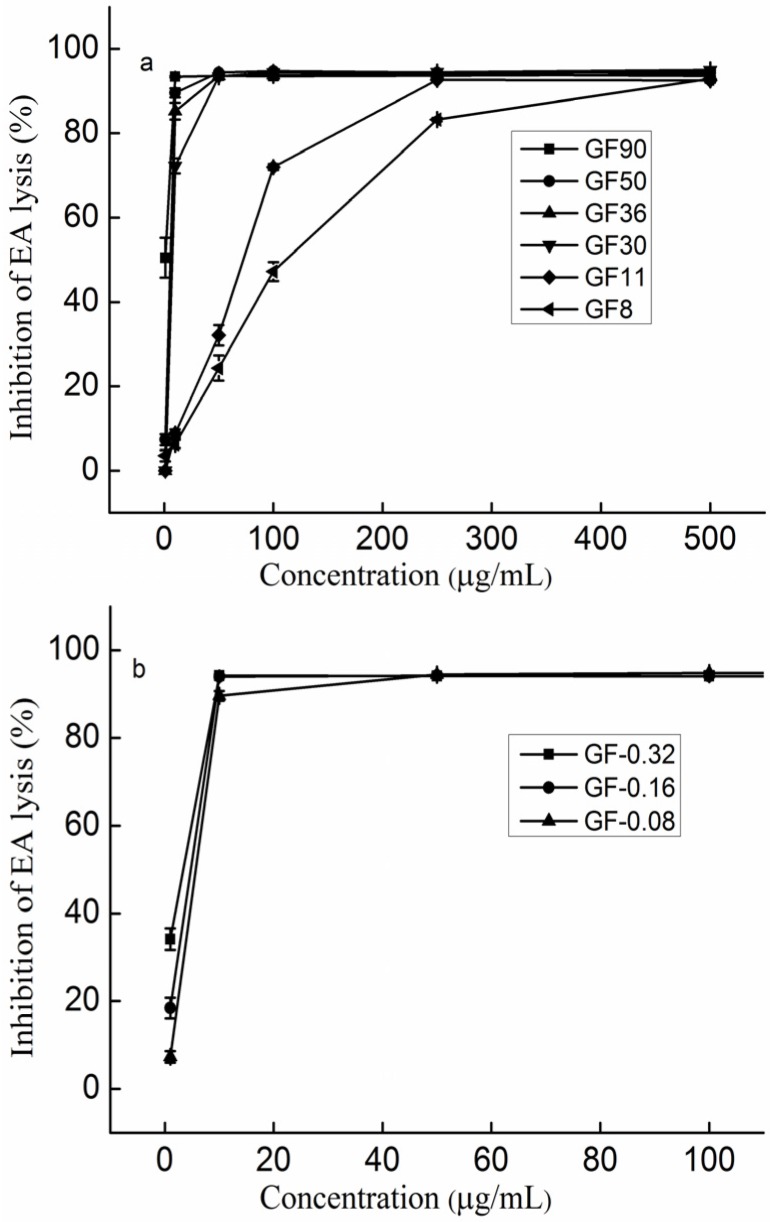
Inhibition of the classical pathway-mediated haemolysis of EA in 1:10-diluted NHS in the presence of increasing amounts of the polysaccharides (**a**) The different molecular weights of sulfated galactofucan; (**b**) Sulfated galactofucan with different molar ratios of galactose to fucose. The results are expressed as percent inhibition of haemolysis. Data are the means from three determinations ± S.E.M. Six sulfated galactofucans weighed 90kDa (GF90), 50kDa (GF50), 36kDa (GF36), 30kDa (GF30), 11kDa (GF11), and 8kDa (GF8). Three sulfated galactofucans had a molar ratio of galactose to fucose of 0.32 (GF-0.32), 0.16 (GF-0.16) and 0.08 (GF-0.08).

To study the relationship between the molecular weight and the anti-complement activity of sulfated galactofucan, six sulfated galactofucans (GF90, GF50, GF36, GF30, GF11, and GF8) (the molar ratio of galactose to fucose was approximately 0.08) with different molecular weights were determined. As described in the previous study [30], six sulfated galactofucans, namely GF90, GF50, GF36, GF30, GF11, and GF8, were prepared. The molecular weights of these were 90.1, 50.1, 36.0, 29.5, 11.3, and 8.4 kDa, respectively. It is shown in Figure 2a that the activity increased as the molecular weights increased. To a certain extent, these results were consistent with previous studies [13,30]. Blondin *et al.* [13] reported that the anti-complement activities of sulfated fucan in the classical pathway increased with increasing molecular weight and reached a plateau at 40 kDa. In addition, the activities also showed dose dependence. GF90 and GF50 had the highest activities at a concentration of 50 µg/mL, while GF8 did not reach the highest activities even at a concentration of 400 µg/mL. Therefore, it was concluded that the molecular weight constituted an important positive factor on the activities.

### 2.4. The Effect of the Molar Ratio of Galactose to Fucose on the Anti-Complement Activity

The chemical compositions of GF-0.32, GF-0.16 and GF-0.08 (also sulfated galactofucans) were reported in a previous study [30]. It was shown that GF-0.32, GF-0.16, and GF-0.08 had similar chemical compositions, except for the molar ratio of galactose to fucose. GF-0.32 weighed 49.3 kDa with a molar ratio of galactose to fucose of 0.32; GF-0.16 weighed 49.2 kDa with 0.16, and GF-0.08 weighed 50.1 kDa with 0.08. Figure 2b shows that all samples reached the highest activities at a concentration of 10 µg/mL. There was little difference in the activities of GF-0.32, GF-0.16, and GF-0.08 at a concentration of 1 µg/mL, indicating that the molar ratio of galactose to fucose contributed to the anti-complement activity. In conclusion, the anti-complement activity was attributed to the molar ratio of galactose to fucose.

### 2.5. The Effect of Sulfate on the Anti-Complement Activity

**Figure 3:**
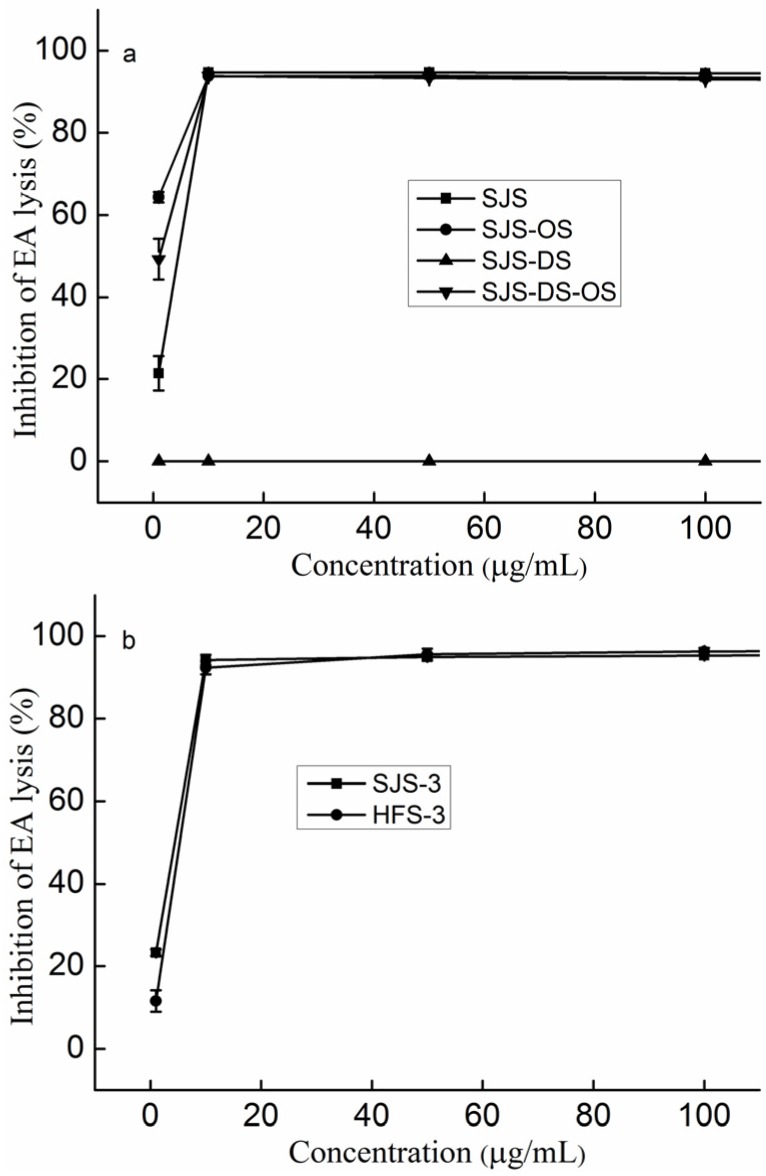
Inhibition of the classical pathway-mediated haemolysis of EA in 1:10-diluted NHS in the presence of increasing amounts of the polysaccharides (**a**) Polysaccharides with different sulfate contents; (**b**) Polysaccharides with different sulfate patterns. The results are expressed as percent inhibition of haemolysis. Data are the means from three determinations ± S.E.M. Overly sulfated SJS (SJS-OS), desulfated SJS (SJS-DS) and desulfated SJS with sulfation (SJS-DS-OS).

Previous studies [31,32,33] have demonstrated the importance of sulfate in the biological activities of polysaccharides. Thus, overly sulfated polysaccharide (SJS-OS), desulfated polysaccharide (SJS-DS) and desulfated polysaccharide with sulfation (SJS-DS-OS) were prepared. Chemical compositions, shown in Table 1, indicated that SJS-OS’s sulfate increased from 36.88% to 46.36%, SJS-DS’s sulfate decreased from 36.88% to 18.12%, and SJS-DS-OS’s sulfate increased from 18.12% to 35.30%. Figure 3a indicates that SJS, SJS-OS and SJS-DS-OS reached the highest activities at a concentration of 10 µg/mL, while SJS-DS had no activity. In addition, SJS-OS had the highest activity at a concentration of 1 µg/mL, SJS-DS-OS second, SJS third and SJS-DS had no activity. It was suggested that a certain sulfate content was necessary for the activities. In addition, the activities did not increase along with increasing sulfate content at a concentration of more than 10 µg/mL.

In addition to the effect of the sulfate content on the activities, the effect of the sulfate pattern was also elucidated by comparison of a pair of sulfated galactofucans (SJS-3 and HFS-3), which were fractionated from SJS and HFS by anion exchange chromatography. According to previous studies [26,28,34,35], SJS-3 is mainly sulfated at C-4 of Fuc, while HFS-3 is mainly sulfated at C-2. The molecular weights of SJS-3 and HFS-3 were 125.4 kDa and 99.2 kDa, respectively, as shown in Table 1, suggesting that the effects of the molecular weight on the activity could be ignored. Moreover, the sulfate content of SJS-3 (38.43%) was similar to HFS-3’s (39.85%). Figure 3b shows the activities of SJS-3 and HFS-3, which were similar to each other at a concentration of more than 10 µg/mL.

Finally, it was concluded that the sulfate content was necessary for the activities; however, the effect of the sulfate on the anti-complement activity was influenced by the concentrations of the samples.

### 2.6. The Effect of the Content of Uronic acid (UA) on the Anti-Complement Activity

**Figure 4:**
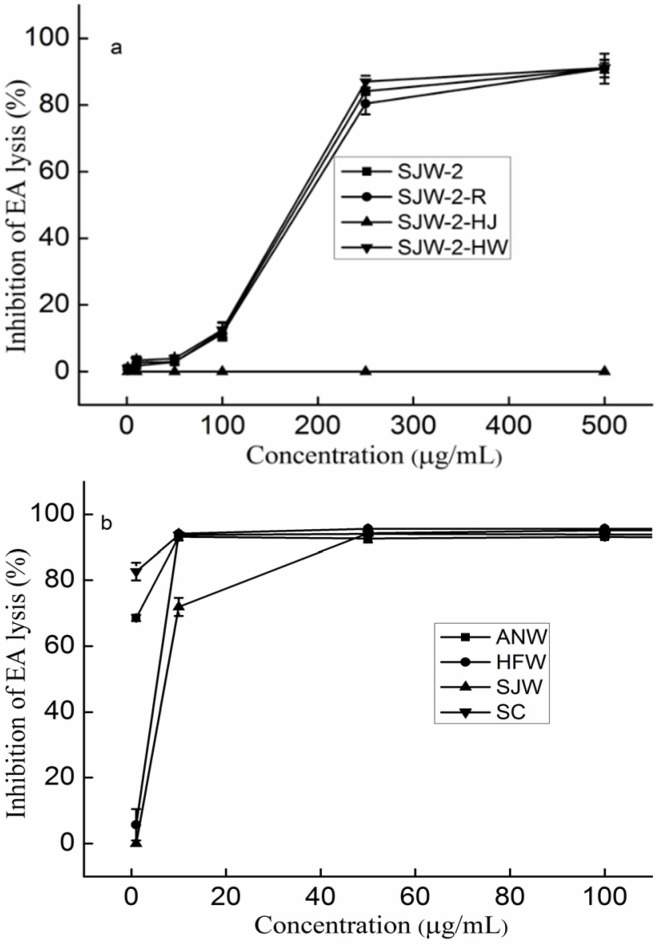
Inhibition of the classical pathway-mediated haemolysis of EA in 1:10-diluted NHS in the presence of increasing amounts of polysaccharides (**a**) Polysaccharides with different linkages; (**b**) Polysaccharides with branching or no branching. The results are expressed as percent inhibition of haemolysis. Data are the means from three determinations ± S.E.M. SJW-2-R was obtained through UA reduction, and SJW-2-HJ and SJW-2-HW were fractionated using an activated carbon column. Polysaccharides derived from *Ascophyllum nodosum* (ANW) and *Acaudina molpadioides* (SC).

From the above results, it was concluded that the anti-complement activities of sulfated galactofucan were better than the heteropolysaccharides with UA. To study the effect of UA content on the activity, three polysaccharides with different UA contents were obtained. One polysaccharide (SJW-2-R) was obtained through UA reduction, and the other two (SJW-2-HJ and SJW-2-HW) were fractionated using an activated carbon column. After reduction, the most apparent changes were in the UA content, which was decreased from 17.35% to 9.86%, while the molar ratio of glucose (Glc) to Fuc was increased from 0 to 0.10, and the glucuronic acid (GlcA) to Fuc ratio decreased from 0.18 to 0.06. Other chemical compositions, shown in Table 1, did not change as obviously. A comparison of the activities of SJW-2 and SJW-2-R in Figure 4a indicates that the change in UA content did not influence the activity. SJW-2-HJ, which was eluted with ethanol, contained a higher UA content and lower sulfate content than that of SJW-2-HW, which was eluted with water. Moreover, SJW-2-HJ had a smaller molecular weight and a higher content of other monosaccharides. Figure 4a shows that SJW-2-HJ did not have any activity, while SJW-2-HW had better activities than that of SJW-2.

In summary, the UA fraction had no anti-complement activity; however, it could reduce the activity in other samples by decreasing the effective concentration of the active compositions when it was mixed with the active compositions. This might explain the observed phenomenon that the heteropolysaccharides with high UA content had lower activities.

### 2.7. The Effect of the Linkage of Sulfated Galactofucan on the Anti-Complement Activity

Crude polysaccharide (ANW) was extracted from brown algae *Ascophyllum nodosum*. According to previous studies [36,37,38,39], it has a backbone of alternating α (1 → 3) and α (1 → 4)-L-Fucp residues and is sulfated at C-2 and possibly C-3 or C-4. SJW contains mainly α (1 → 3)-L-Fucp residues sulfated at C-4, while HFW is mainly composed of α (1 → 3)-L-Fucp residues sulfated at C-2 [26,27]. Figure 4b shows the anti-complement activities of ANW, SJW, and HFW. They all showed dose dependence and reached a plateau. Specifically, the IC_50_ values of ANW, SJW, and HFW were 0.98, 7.26, and 5.51 µg/mL, respectively. The differences in the activities could be explained by noting that SJW had a higher UA content, while HFW had a higher content of laminaran, which had no anti-complement activity (as discussed in Section 3.9). Thus, it was proposed that there might be little or no effect of the linkage on the anti-complement activity.

### 2.8. The Effect of the Branching of Polysaccharides on the Anti-Complement Activity

Polysaccharide (SC) was extracted from sea cucumber *Acaudina molpadioides* according to a previous study [40]. It was reported [16] that polysaccharides from marine invertebrates possess a clearly linear backbone, consisting of a regular repeating unit, in contrast with the algal polysaccharides. Thus, the anti-complement activities of the polysaccharides from algae and marine invertebrates were determined to elucidate the effect of the branching. The activity of SC in Figure 4b is similar to that of ANW, indicating that there was little impact of branching on the anti-complement activities. This result contrasts a previous study [41] that suggested that branching of fucoidan oligosaccharides had a major impact on their anti-complement activity. This difference might be explained based on the conformational state of the polysaccharides. The conformational states of the oligosaccharides depend on branching, while the conformational states of the polysaccharides do not. Therefore, it was concluded that there might be little or no effect of the branching of polysaccharides on the anti-complement activity.

### 2.9. The Effect of the Type of Monosaccharides on the Anti-Complement Activity

**Figure 5:**
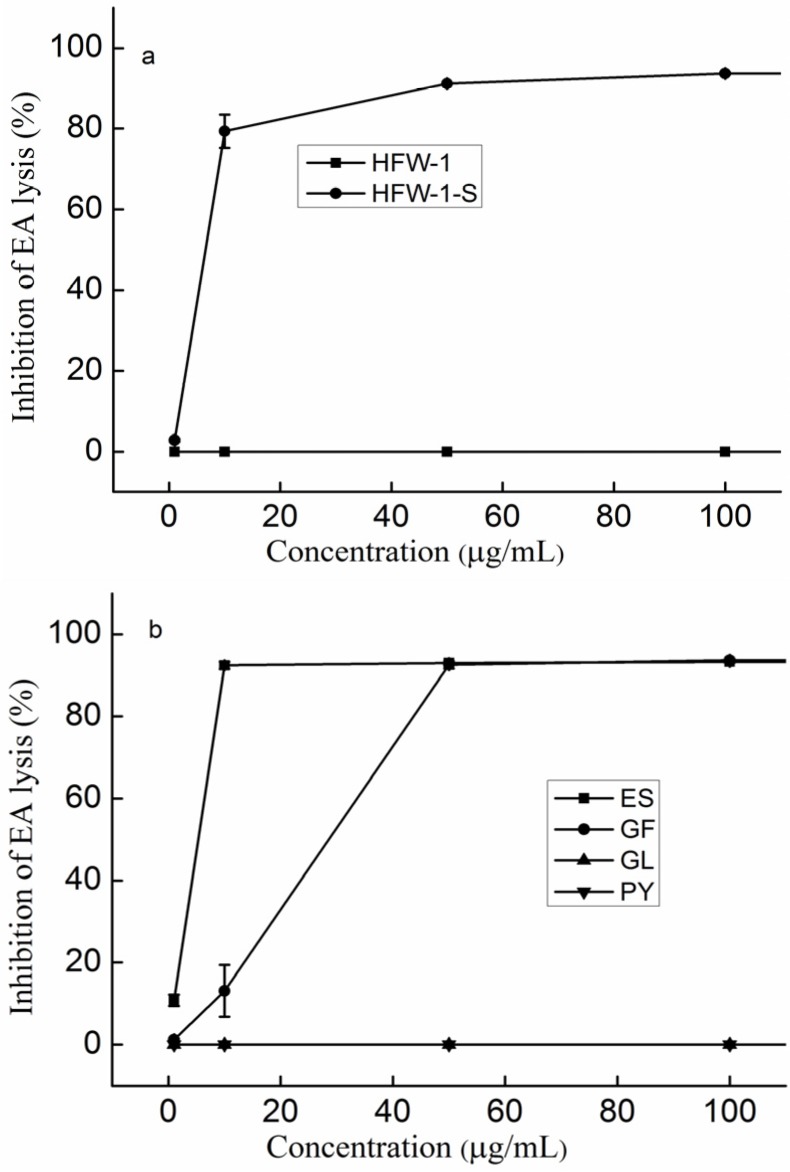

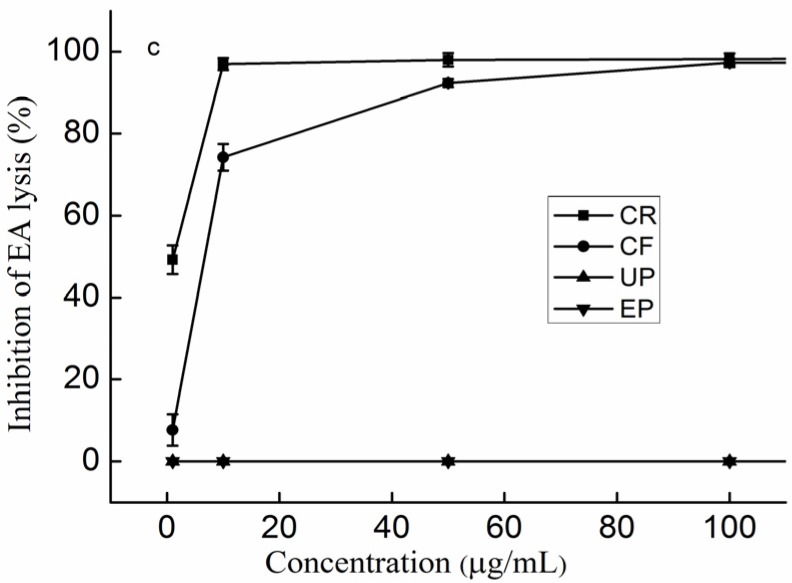
Inhibition of the classical pathway-mediated haemolysis of EA in 1:10-diluted NHS in the presence of increasing amounts of the polysaccharides (**a**) Laminaran and sulfated laminaran; (**b**) Polysaccharides from red algae; (**c**) Polysaccharides from green algae. The results are expressed as percent inhibition of haemolysis. Data are the means from three determinations ± S.E.M. Polysaccharides derived from *Eucheuma spinosum* (ES), *Grateloupia filicina* (GF), *Gracilaria lamaneiformis* (GL), *Porphyra yezoensis* (PY), *Enteromorpha prolifera* (EP), *Ulva pertusa* (UP), *Cladophera rupestris* (CR) and *Codium fragile* (CF). SJW-2-R was obtained through UA reduction, and SJW-2-HJ and SJW-2-HW were fractionated using an activated carbon column. Overly sulfated laminaran (HFW-1-S).

To confirm whether Fuc was necessary for the activity, sulfated laminaran (HFW-1-S) was prepared. Figure 5a shows that laminaran (both HFW-1 and HFS-1 were laminaran) had no activity, while sulfated laminaran had good activity, suggesting that Fuc was not necessary for the activity. Thus, it was concluded that laminaran, like the UA fraction, had no activity. However, it could reduce the activity by decreasing the effective concentrations of the active components. In addition, it also confirmed that the fucose residue was not necessary.

Four types of crude polysaccharides from red algae, namely *Grateloupia filicina* (GF), *Eucheuma spinosum* (ES), *Gracilaria lamaneiformis* (GL), and *Porphyra yezoensis* (PY), were prepared. ES is a carrageenan polysaccharide; GF is an agar-carrageenan intermediate polysaccharide, and GL and PY are agar polysaccharides. The most apparent difference in the chemical compositions, as shown in Table 1, is the sulfate content. Figure 5b shows that ES had the best anti-complement activity, GF second, and GL and PY had no activities, indicating that sulfate was essential for the anti-complement activity. It was also confirmed that Fuc was not necessary.

Four types of crude polysaccharides from green algae, namely *Cladophera rupestris* (CR), *Codium fragile* (CF), *Enteromorpha prolifera* (EP), and *Ulva pertusa* (UP), were prepared. The analysis of the chemical compositions in Table 1 indicates that the former two belonged to type 1, while the latter two were type 2. Figure 5c shows that CR and CF showed good anti-complement activities, while EP and UP did not. This might be explained by the high content of UA. In addition, the solution of the polysaccharides with high UA content was too thick.

The main polysaccharides from brown algae (*Saccharina japonica* and *Hizikia fusiforme*) were laminaran, alginate, and fucoidan. Fucoidan might be classified as a combination of sulfated heteropolysaccharides and sulfated galactofucan. In the above results, it was concluded that sulfated galactofucan was the active component of the anti-complement activity. In addition, the results also showed that laminaran did not have any activity. Moreover, it was indicated that heteropolysaccharides with UA had lower activity. Alginate (data not shown) displayed no activity, which might confirm this.

Thus, it was concluded that the types of monosaccharides (neutral monosaccharides) had no effect on the anti-complement activity.

## 3. Experimental Section

### 3.1. Materials

The brown algae *Saccharina japonica* was collected in Rongcheng, Qingdao, China, on 28 May 2014. The brown algae *Hizikia fusiforme* was purchased from Dong Tou, Zhejiang, China, on 18 July 2014. The green algae *Enteromorpha prolifera* was collected in Qingdao, China, on 15 June 2012. The green algae *Codium fragile* and *Ulva pertusa* were collected in Qingdao, China, on 12 December 2013. The green algae *Cladophera rupestris* was collected in Rongcheng, Qingdao, China, on 24 July 2014. The red algae *Porphyra yezoensis* was purchased from Lianyungang, Jiangsu, China. The red algae *Grateloupia filicina*, *Eucheuma spinosum,* and *Gracilaria lamaneiformis* were purchased from Zhanjiang, China. *Ascophyllum nodosum* and *Acaudina molpadioides* were purchased from Qingdao, China. These were authenticated by Professor Zuhong Xu.

### 3.2. Extraction and Preparation of Polysaccharides

Brown algae (*Saccharina japonica, Hizikia fusiforme*, and *Ascophyllum nodosum*) (100 g) were cut into pieces and treated with 85% ethanol three times to remove the pigment. Crude polysaccharide was extracted from the residual material with hot water (3 L) for 4 h. The extract solution was filtered with Celite and concentrated. Further elimination of algin was achieved using 20% ethanol with MgCl_2_ (0.05 M/L). After removing the algin, the supernatant fluid was ultra-filtered. Finally, the dialysate was concentrated, and the crude polysaccharides were obtained by ethanol precipitation and named SJW, HFW, and ANW, respectively.

Green algae and red algae (100 g) were cut into pieces and treated with 85% ethanol three times to remove the pigment. Crude polysaccharides were extracted from the residual material with hot water (3 L) for 4 h. The extract solution was filtered with Celite, concentrated, and dialyzed. Finally, the dialysate was concentrated, and the crude polysaccharides were obtained by ethanol precipitation.

Crude polysaccharides from brown algae (*Saccharina japonica* and *Hizikia fusiforme*) were extracted from the residual material with 0.1 M HCl (2 L) at room temperature for 3 h. The extract solution was filtered with Celite, ultra-filtered, and concentrated. Finally, the crude polysaccharides were obtained by ethanol precipitation and named SJS and HFS.

Crude polysaccharide (SC) was extracted from the sea cucumber *Acaudina molpadioides* as previously described [40]. Briefly, crude polysaccharide was extracted from the fresh body wall (10 g) with hot water (500 mL) for 4 h. The extract solution was filtered with Celite and concentrated. The concentrated solution was hydrolysed with papain. Papain was deactivated by hot water. Then, the solution was centrifuged, concentrated, and dialyzed. Finally, the dialysate was concentrated, and the crude polysaccharide was obtained by ethanol precipitation and named SC.

### 3.3. Preparation and Purification of Polysaccharides

Crude polysaccharides (SJW and SJS) (8 g) underwent anion exchange chromatography on a DEAE-Bio Gel Agarose FF gel (6 cm × 40 cm) with elution by 0.5 M (5 L) (SJW-1 and SJS-1), 1 M (5 L) (SJW-2 and SJS-2), and 2 M NaCl (5 L) (SJW-3 and SJS-3). The polysaccharides were then ultrafiltrated, concentrated, and precipitated by ethanol.

Crude polysaccharides (HFW and HFS) (8 g) also underwent anion exchange chromatography on a DEAE-Bio Gel Agarose FF gel (6 cm × 40 cm) with elution by water (5 L) (HFW-1 and HFS-1), 0.5 M (5 L) (HFW-2 and HFS-2), and 2 M NaCl (5 L) (HFW-3 and HFS-3). The polysaccharides were then ultrafiltrated, concentrated, and precipitated by ethanol.

Fraction SJW-2 was fractionated using an activated carbon column (2.6 cm × 30 cm) with elution by water (1 L) (SJW-2-HW) and a gradient elution from 50% ethanol to 95% ethanol (SJW-2-HJ). The polysaccharides were concentrated and precipitated by ethanol.

### 3.4. Preparation of Desulfated Polysaccharides

The desulfated polysaccharides were prepared according to a method reported in the literature [27]. Briefly, SJS (1 g) was dissolved in distilled water (100 mL) and mixed with cationic resin (H^+^) for 3 h. After filtration, the solution was neutralized with pyridinium and lyophilized. The solution was dissolved in dimethyl sulfoxide (DMSO): methanol (9:1; *v*/*v*, 20 mL). The mixture was heated at 80 °C for 5 h, and the desulfated products (SJS-DS) were dialyzed and lyophilized.

### 3.5. Preparation of over-Sulfated Polysaccharides

The over-sulfated polysaccharides were prepared according to a method reported in the literature [42]. Briefly, polysaccharides (SJS, SJS-DS and HFW-1) (1 g) in dimethyl formamide (DMF) (20 mL) were treated with a sulphur trioxide pyridine complex (5 g) at 50 °C for 24 h, and then the over-sulfated products (SJS-OS, SJS-DS-OS and HFW-1-S) were neutralized, dialyzed, and lyophilized.

### 3.6. Preparation of the Polysaccharides with Carboxyl Reduction

Carboxyl reduction was performed as previously described [43]. Briefly, SJW-2 (1 g) was dissolved in distilled water (50 mL), followed by the addition of 1-ethyl-3-(3-dimethyl-aminopropyl) carbodiimide (EDC). The solution was stirred at room temperature for 1 h under constant pH conditions (approximately 4.8) by the addition of 0.1 M HCl. Then, freshly prepared 2 M sodium borohydride (10 mL) was added twice during the next 2 h at 50 °C. Later, the reaction was terminated by the addition of glacial acetic acid. Finally, the polysaccharide with carboxyl reduction (SJW-2-R) was dialyzed, concentrated, and lyophilized.

### 3.7. Compositional Analysis

The sulfated contents were performed by ion chromatography on a Shodex IC SI-52 4E column (4.0 × 250 mm) and eluted with 3.6 mM Na_2_CO_3_ at a flow rate of 0.8 mL/min at 45 °C. The molar ratios of monosaccharides and fucose content were determined as described by Zhang *et al.* [44]. The uronic acid (UA) concentration was determined by a modified carbazole method [45]. The molecular weights of the polysaccharides were evaluated by GPC-HPLC on a TSK G3000 PWxl column (7 μm 7.8 × 300 mm) with elution in 0.05 M Na_2_SO_4_ at a flow rate of 0.5 mL/min at 40 °C with refractive index detection. Ten different molecular weight dextrans purchased from the National Institute for the Control of Pharmaceutical and Biological Products (Beijing, China) were used as weight standards.

### 3.8. Anti-Complement Activity

According to previous studies [4,46], the anti-complement activities of the polysaccharides were determined following the classical pathway. In the classical pathway, various dilutions (100 μL) of the polysaccharides were mixed with 1:10 diluted normal human serum (NHS, which was obtained from healthy adult donors) (100 μL), GVB^2+^ (veronal buffer saline [VBS] containing 0.1% gelatin, 0.5 mM Mg^2+^ and 0.15 mM Ca^2+^) (200 μL) and sensitized erythrocytes (EA) (200 μL). Then, the mixture was incubated at 37 °C for 30 min. The following assay controls were incubated under the same conditions: (1) 100% lysis: EA (200 μL) in water (400 μL); (2) sample control: sample (100 μL) in GVB^2+^ (500 μL); (3) complement: 1:10-diluted NHS (100 μL) and EA (200 μL) in GVB^2+^ (300 μL); and (4) blank: EA (200 μL) in GVB^2+^ (400 μL). After incubation, the mixture was centrifuged (5000 rpm × 10 min) and the erythrocyte lysis was determined at 405 nm. Decreased lysis in the presence of tested polysaccharides indicated anti-complement activity. All of the samples were dissolved in GVB^2+^. The percent inhibition was calculated using the following equation: inhibition of EA lysis (%) = (A_complement_ − (A_sample_ − A_sample control_))/A_complement_ × 100.

### 3.9. Statistical Analysis

All data are shown as the mean ± standard deviation (SD). Significant differences between the experimental groups were determined by one-way ANOVA, and differences were considered to be statistically significant if *p* < 0.05. All calculations were performed using SPSS 16.0 statistical software.

## 4. Conclusions

In summary, four crude polysaccharides (SJW, SJS, HFS, and HFW) were extracted by water and 0.1 M HCl from *Saccharina japonica* and *Hizikia fusiforme* to determine the effects of the extraction methods. The results could not definitively elucidate the effect of the extraction method. Crude polysaccharides were fractionated using anion exchange chromatography. It was found that sulfated galactofucan maintained its activity, indicating that it was the active composition. To study the effect of molecular weight, several different molecular weight polysaccharides were obtained. It was shown that the larger the molecular weight, the better the activity. However, the activity reached a plateau. In addition, the effect of the molar ratio of galactose to fucose was also considered. It was observed that the molar ratio of galactose to fucose was a positive factor. Moreover, the activities of SJS-OS, SJS-DS, and SJS-DS-OS confirmed that sulfate was an important positive factor. The activities of HFS-3 and SJS-3 suggested that the sulfation pattern had no effect at a concentration of more than 10 µg/mL, like linkage and branching. The type of monosaccharides had no effect. Finally, it was concluded that the UA fraction and laminaran had no effect on the anti-complement activity; however, it could reduce the activity by decreasing the effective concentration of the active composition.

## References

[1] Sahu A., Lambris J.D. (2000). Complement inhibitors: A resurgent concept in anti-inflammatory therapertics. Immunopharmacology.

[2] Morgan B.P., Harris C.L. (2003). Complement therapertics: History and current progress. Mol. Immunol..

[3] Maillet F., Maurice P., Jean C., Kazatchkine M.D. (1988). Structure-function relationships in the inhibitory effect of heparin on complement activation: Independency of the anti-coagulant and anti-complementary sites on the heparin molecule. Mol. Immunol..

[4] Xu H., Zhang Y., Zhang J., Chen D. (2007). Isolation and characterization of an anti-complementary polysaccharide D3-S1 from the roots of *Bupleurum smithii*. Int. Immunopharmacol..

[5] Fang X.B., Yin X.X., Yuan G.F., Chen X.O. (2015). Chemical and biological characterization of polysaccharides from the bark of *Avicennia marina*. Eur. Food Res. Technol..

[6] Samuelsena A.B., Lunda I., Djahromia J.M., Paulsena B.S., Wolda J.K., Knutsen H.S. (1999). Structural features and anti-complementary activity of some heteroxylan polysaccharide fractions from the seeds of *Plantago major* L.. Carbohydr. Polym..

[7] Ni F., Liu L., Song Y., Wang X., Zhao Y., Huang W., Wang Z., Xiao W. (2015). Anti-complementary phenolic acids from *Lonicera japonica*. China J. Chin. Mater. Med..

[8] Sun Q., Bao J. (2015). Purification, cloning and characterization of a metalloproteinase from *Naja atra* venom. Toxicon.

[9] Makrides S.C. (1998). Therapeutic Inhibition of the Complement System. Pharmacological.

[10] Xu X., Chen L., Zhao X. (2015). Review on the research and development of anti-complementary agents from natural products. Nat. Prod. Res. Dev..

[11] Mauzac M., Maillet F., Jozefonvicz J., Kazatchkine M.D. (1985). Anticomplementary activity of dextran derivatives. Biomaterials.

[12] Crepon B., Maillet F., Kazatchkine M.D., Jozefonvicz J. (1987). Molecular weight dependency of the acquired anticomplementary and anticoagulant activities of specifically substituted dextrans. Biomaterials.

[13] Blondin C., Chaubet F., Nardella A., Sinquin C., Jozefonvicz J. (1996). Relationships between chemical characteristics and anticomplementary activity of fucans. Biomaterials.

[14] Zhang W., Jin W., Sun D., Zhao L., Wang J., Duan D., Zhang Q. (2015). Structural analysis and anti-complement activity of polysaccharides from *Kjellmaniella crsaaifolia*. Mar. Drugs.

[15] Blondin C., Fisher E., Boisson-Vadal C., Kazatchkine M.D., Jozefonvicz J. (1994). Inhibition of complement activation by natural sulfated polysaccharides (fucans) from brown seaweed. Mol. Immunol..

[16] Berteau O., Mulloy B. (2003). Sulfated fucans, fresh perspectives: Structures, functions, and biological properties of sulfated fucans and an overview of enzymes active toward this class of polysaccharide. Glycobiology.

[17] Bilan M.I., Grachev A.A., Shashkov A.S., Kelly M., Sanderson C.J., Nifantiev N.E., Usov A.I. (2010). Further studies on the composition and structure of a fucoidan preparation from the brown alga *Saccharina latissima*. Carbohydr. Res..

[18] Jiao G., Yu G., Zhang J., Ewart H. (2011). Chemical structures and bioactivities of sulfated polysaccharides from marine algae. Mar. Drugs.

[19] Kusaykin M., Bakunina I., Sova V., Ermakova S., Kuznetsova T., Besednova N., Zaporozhets T., Zvyagintseva T. (2008). Structure, biological activity, and enzymatic transformation of fucoidans from the brown seaweeds. Biotechnol. J..

[20] Li B., Lu F., Wei X., Zhao R. (2008). Fucoidan: Structure and Bioactivity. Molecules.

[21] Pomin V.H., Mourao P.A.S. (2008). Structure, biology, evolution, and medical importance of sulfated fucans and galactans. Glycobiology.

[22] Lahaye M. (2001). Developments on gelling algal galactans, their structure and physico-chemistry. J. Appl. Phycol..

[23] Pomin V.H. (2010). Structural and functional insights into sulfated galactans: A systematic review. Glycoconj. J..

[24] USov A.I. (1998). Structural analysis of red seaweed galactans of agar and carrageenan groups. Food Hydrocoll..

[25] Lahaye M., Robic A. (2007). Structure and Functional Properties of Ulvan, a Polysaccharide from Green Seaweeds. Biomacromolecules.

[26] Jin W., Zhang W., Wang J., Ren S., Song N., Duan D., Zhang Q. (2014). Characterization of laminaran and a highly sulfated polysaccharide from *Sargassum fusiforme*. Carbohydr. Res..

[27] Jin W., Zhang W., Wang J., Ren S., Song N., Zhang Q. (2013). Structural analysis of heteropolysaccharide from *Saccharina japonica* and its derived oligosaccharides. Int. J. Biol. Macromol..

[28] Jin W., Guo Z., Wang J., Zhang W., Zhang Q. (2013). Structural analysis of sulfated fucan from *Saccharina japonica* by electrospray ionization tandem mass spectrometry. Carbohydr. Res..

[29] Jin W., Wang J., Ren S., Song N., Zhang Q. (2012). Structural analysis of a heteropolysaccharide from *Saccharina japonica* by electrospray mass spectrometry in tandem with collision-induced dissociation tandem mass spectrometry (ESI-CID-MS/MS). Mar. Drugs.

[30] Jin W., Zhang Q., Wang J., Zhang W. (2013). A comparative study of the anticoagulant activities of eleven fucoidans. Carbohydr. Polym..

[31] Cumashi A., Ushakova N.A., Preobrazhenskaya M.E., D’Incecco A., Piccoli A., Totani L., Tinari N., Morozevich G.E., Berman A.E., Bilan M.I. (2007). A comparative study of the anti-inflammatory, anticoagulant, antiangiogenic, and antiadhesive activities of nine different fucoidans from brown seaweeds. Glycobiology.

[32] Karmakar P., Pujol C.A., Damonte E.B., Ghosh T., Ray B. (2010). Polysaccharides from *Padina tetrastromatica*: Structural features, chemical modification and antiviral activity. Carbohydr. Polym..

[33] Pereira M.S., Melo F.R., Mourão P.A.S. (2002). Is there a correlation between structure and anticoagulant action of sulfated galactans and sulfated fucans?. Glycobiology.

[34] Li B., Xue X.J., Sun J.L., Xu S.Y. (2006). Structural investigation of a fucoidan containing a fucose-free core from the brown seaweed *Hizikia fusiforme*. Carbohydr. Res..

[35] Wang P., Zhao X., Lv Y., Liu Y., Lang Y., Wu J., Liu X., Li M., Yu G. (2012). Analysis of structural heterogeneity of fucoidan from *Hizikia fusiforme* by ES-CID-MS/MS. Carbohydr. Polym..

[36] Chevolot L., Foucault A., Chaubet F., Kervarec N., Sinquin C., Fisher A.M., Boisson-Vidal C. (1999). Further data on the structure of brown seaweed fucans: Relationships with anticoagulant activity. Carbohydr. Res..

[37] Chevolot L., Mulloy B., Ratiskol J., Foucault A., Colliec-Jouaultb S. (2001). A disaccharide repeat unit is the major structure in fucoidans from two species of brown algae. Carbohydr. Res..

[38] Daniel R., Berteau O., Jozefonvicz J., Goasdoue N. (1999). Degradation of algal *(Ascophyllum nodosum*) fucoidan by an enzymatic activity contained in digestive glands of the marine mollusc Pecten maximus. Carbohydr. Res..

[39] Daniel R., Berteau O., Chevolot L., Varenne A., Gareil P., Goasdoue N. (2001). Regioselective desulfation of sulfated l-fucopyranoside by a new sulfoesterase from the marine mollusk *Pecten maximus*. Application to the structural study of algal fucoidan (*Ascophyllum nodosum*). Eur. J. Biochem..

[40] Xue C., Tang Q., Li D., Wu X., Wang J. (2010). Isolation and characterization of a sea cucumber fucoidan-utilizing marine bacterium. Lett. Appl. Microbiol..

[41] Clement M.J., Tissot B., Chevolot L., Adjadj E., Du Y., Curmi P.A., Daniel R. (2010). NMR characterization and molecular modeling of fucoidan showing the importance of oligosaccharide branching in its anticomplementary activity. Glycobiology.

[42] Nifantiev N., Ustyuzhanina N., Krylov V., Grachev A., Gerbst A. (2006). Synthesis, NMR and conformational studies of fucoidan fragments, 8: Convergent synthesis of branched and linear oligosaccharides. Synthesis.

[43] Kariya Y., Watabe S., Kyogashima M., Ishihara M., Ishii T. (1997). Structure of fucose branches in the glycosaminoglycan from the body wall of the sea cucumber *Stichopus japonicus*. Carbohydr. Res..

[44] Zhang J., Zhang Q., Wang J., Shi X., Zhang Z. (2009). Analysis of the monosaccharide composition of fucoidan by precolumn derivation HPLC. Chin. J. Oceanol. Limnol..

[45] Bitter T., Muir H.M. (1962). A modified uronic acid carbazole reaction. Anal. Biochem..

[46] Klerx J.P.A.M., Beukelman C.J., Dijk H.V., Willers J.M.N. (1983). Microassay for colorimetric estimation of complement activity in guinea pig, human and mouse serum. J. Immunol. Methods.

